# Microglial expression of Alzheimer’s risk factor BIN1 regulates tau pathology in PS19 mice

**DOI:** 10.1002/alz.087071

**Published:** 2025-01-03

**Authors:** Ari Sudwarts, Mitchell Hansen, Shuai Wang, Stanislau Smirnou, Vlada Skorobovenko, Michaela Delozier, Gopal Thinakaran

**Affiliations:** ^1^ Byrd Alzheimer’s Center and Research Institute, Tampa, FL USA; ^2^ Byrd Alzheimer’s Center & Research Institute, Tampa, FL USA

## Abstract

**Background:**

Microglia play significant roles in Alzheimer’s disease (AD) pathophysiology. Current evidence suggests microglia may function in both protective and degenerative capacities, which has received little clarity from transcriptionally‐characterised phenotypes uncovered from transgenic pathologies alone. *BIN1* ‐ the second‐most significant risk gene for the development of late‐onset AD (LOAD) ‐ is expressed at high levels in neurons, oligodendrocytes and microglia. We examined microglial BIN1 expression and function and previously demonstrated that BIN1 regulates proinflammatory and disease‐associated activation responses in microglia in vitro and in vivo (PMID 35526014). However it’s role in AD‐specific pathologies is as yet unknown.

**Method:**

We used a reverse‐genetic approach to conditionally delete *Bin1* in microglia of the PS19 mouse model of tauopathy. We used histology, biochemistry and RNAseq analyses to determine the effects of *Bin1*‐cKO on tau pathology and microglial responses.

**Result:**

Our data demonstrate that microglial BIN1 facilitates levels of pathologically phosphorylated tau (p‐tau), specifically in female PS19 mice. RNAseq analysis identified cell ‐autonomous and non‐autonomous effects for microglial BIN1 during tau pathogenesis in the PS19 model. Weighted gene co‐expression analyses revealed complex networks of genes regulated by microglial gene expression during tau pathogenesis. We also identified gene networks correlated with levels of formic acid‐soluble p‐tau, irrespective of microglial *Bin1* gene manipulation.

**Conclusion:**

These gene networks offer novel insight into microglial genes involved in responses to tau pathology, and the potential impact of *BIN1* as a LOAD risk gene.

